# Modeling, Control, and Optimization in Aeronautical Engineering

**DOI:** 10.1155/2015/979107

**Published:** 2015-06-15

**Authors:** Zheng Zheng, Kemao Peng, Wenbo Du, Guangquan Zhang

**Affiliations:** ^1^School of Automation Science and Electrical Engineering, Beihang University, Beijing 100191, China; ^2^Temasek Laboratories, National University of Singapore, Singapore; ^3^School of Electronic and Information Engineering, Beihang University, Beijing 100191, China; ^4^Centre for Quantum Computation and Intelligent Systems, University of Technology, Sydney, NSW 2007, Australia

The last decade has witnessed the rapid development of aeronautical engineering including all branches of applied sciences and technology dealing with aircraft and their support systems, which brings forward the higher request of safety, efficiency, and environmental protection. Modeling, control, and optimization of aeronautical engineering have played an increasingly important role in meeting aeronautical requirements, and they have drawn widespread attention from communities including control theory, intelligent optimization, system science, real-time distributed computing, electronic information engineering, and aeronautical engineering industry. Driven by such motivations, the main focus of this special issue is on the new theories, new technologies, and their applications in modeling, control, and optimization for aeronautical engineering systems.

The special issue is a collection of original research articles, whose authors and editors belong to academic or research institutions of five different countries from Asia, Europe, and Australia. The full papers in this issue can be broadly organized into two main categories: (i) modelling and optimization and (ii) control techniques.

Papers in category (i) are mainly concerned with network optimization of air transportation system and terminal-area operation. Recently, people come to realize that the air transportation system can be modeled as complex networks. Along this line, K. Cai et al. in “A Novel Biobjective Risk-Based Model for Stochastic Air Traffic Network Flow Optimization Problem” and X. Guan et al. in “An Airway Network Flow Assignment Approach Based on an Efficient Multiobjective Optimization Framework” modeled the flight operation as network flow optimization problem and proposed novel algorithms, respectively. Terminal-area operation is the bottleneck of the air transportation system. Y. Yang et al. investigated the aircraft intent inference approach and proposed an online trajectory clustering method, which will be meaningful to the efficiency and safety of busy terminal area.

Papers in category (ii) are focusing on the topics of rotary-wing aircrafts and robots. The rotary-wing aircraft is one of the popular platforms in the control community such as the helicopter and quadrotor. T. Oktay and F. Sal in “Helicopter Control Energy Reduction Using Moving Horizontal Tail” attempted to improve the flight duration by reducing consumption of the energy. X. Xu et al. in “MRAC Control with Prior Model Knowledge for Asymmetric Damaged Aircraft” intended to explore control of the wing-damaged aircraft, which is one of advanced topics to improve survivability of the fighters. Z. Li and Y. Wang in “Coordinated Control of Slip Ratio for Wheeled Mobile Robots Climbing Loose Sloped Terrain” attempted to explore the control of mobile robots in 3D environments. J. López et al. in “A Robust *H*_*∞*_ Controller for an UAV Flight Control System” implemented and validated a robust *H*_*∞*_ controller for an UAV to track different types of manoeuvres in the presence of noisy environment.

It should be mentioned that a special issue simply provides a snapshot of the field taken at a particular point in time. Due to the standard page limitations of a journal volume, it can only include a relatively small number of papers. As a result, its coverage is by no means complete despite our best efforts.

